# Maize Stalk Material for On-Site Treatment of Highly Polluted Leachate and Mine Wastewater

**DOI:** 10.3390/ma14040956

**Published:** 2021-02-18

**Authors:** Nicoleta Mirela Marin, Laurentiu Dinu, Ioana Stanculescu, Nicolae Ionut Cristea, Alexandra Ioana Ionescu

**Affiliations:** 1 National Research and Development Institute for Industrial Ecology ECOIND, Street Podu Dambovitei no. 71-73, District 6, 060652 Bucharest, Romania; laurentiu.dinu@incdecoind.ro (L.D.); ionut.cristea@incdecoind.ro (N.I.C.); ioana.ionescu@incdecoind.ro (A.I.I.); 2 Department of Physical Chemistry, Faculty of Chemistry, University of Bucharest, 4-12 Regina Elisabeta Bd., 030018 Bucharest, Romania; 3 Horia Hulubei National Institute for Physics and Nuclear Engineering, Centre of Technological Irradiations IRASM, 30 Aleea Reactorului, 077125 Magurele, Romania

**Keywords:** biomaterial, metals, tailing, outflow, inflow, laboratory pilot experiment

## Abstract

New research applications involving the use of cellulosic material derived from maize stalk for on-site treatment of leachate were evaluated for specific removal of Cu(II) and Fe(III) from real, highly polluted tailing pond and mine wastewater samples. Two major issues generated by anthropic mining activities were also tackled: wastewater metal content decrease to improve water quality and subsequently metal specific recovery, increasing the economic efficiency of metal production by using a green technology for residual management. Rapid saturation of the maize stalk mass determined in batch studies and the mine pilot experiment led to diminished metal concentrations in the second pilot experiment, where Cu(II) and Pb(II) from synthetic solutions were monitored in order to test biomaterial performances. In addition, in the second pilot experiment, maize stalk removed Pb(II) in the first 36 h, below the determination limit of the analytical method. The biomaterial bed in the column was saturated after 252 h of inflow solution. FTIR-ATR, TG and SEM techniques probed the interaction between maize stalk polar groups C=O, –OH, C–O and tailing water metallic ions by large FTIR band displacements, intensity decrease and shape changes, modification of thermal stability and by changes in the appearance of adsorbent microstructure images owing mainly to ion exchange mechanism.

## 1. Introduction

Water pollution with heavy metals, resulting from mining activities, is one of the global risks for human health due to the hydrological cycle and subsequent incorporation into the water supply and food chains. Due to the timely increase in industrialization, serious pollution of ecosystems may be witnessed, especially with heavy metals, including copper, cadmium, lead, arsenic and mercury, which are highly toxic and tend to accumulate in the environment. These metals are poisonous to humans, especially when their concentration is higher than the limits established by the World Health Organization. Currently, the feasibility of using different adsorbents of vegetable origin, called green adsorbents, is tested in the field of wastewater treatments in the search for more efficient depollution means. Green adsorbents are cheap and available in significant quantities as residues of agricultural crop processing. Many researchers have studied eco-friendly technologies to efficiently treat wastewater at sufficiently low cost [1,2,3,4,5,6,7]. One may observe that wastewater metal separation methods involving green adsorbents are simple and economically advantageous. The capacity to separate metallic ions in different concentrations from water sources has been highlighted in different research papers [8,9,10]. Moreover, to improve adsorption capacity, activation and or process modifications may be done with different reagents. However due to their toxicity they may produce negative effect on the environment at the end of adsorption process. Thus, regeneration and reuse of the biomaterial is one important attribute of green adsorbent technologies. The basic components of these biomaterials are cellulose, hemicellulose and lignin, as well as other compounds in smaller proportions, including lipids and proteins. Consequently, the functional group of these compounds proves affinity for wastewater metallic ions that may be retained by various mechanisms such as ion exchange or inside pores by diffusion [11]. Moreover, in recent years, many natural potential bio-sorbents suitable for metal removal using batch mode from aqueous solutions have been reported in the literature [12,13,14,15,16,17,18,19,20,21,22,23]. Chemical modification improves the adsorption capacity of cellulosic material. Therefore, chemical transformation was done directly on the surface of the bio-adsorbent, and new functional groups were attached to the basic components [12,13]. Zeng et al. have studied Cd(II) adsorption from aqueous solution using raw corn stalk modified with acrylonitrile. The modified stalk was characterized by IR and CP/MAS 13 NMR. The maximum adsorption capacities were detected as 12.73 and 3.39 mg/g for the modified and unmodified stalk, respectively [12]. Moreover, cellulose was isolated from raw corn stalk and subsequently modified by graft polymerization. The obtained results showed that modified cellulose had better adsorption potential for Cd(II) than unmodified cellulose because new functional groups of –CN and –OH were added [13]. Vaughan et al. used corn cobs to retain cadmium, copper, lead, nickel and zinc from 20-mM synthetic solutions. The corn stalk was modified with citric acid (0.6 M) or phosphoric acid (1 M) [15]. Dried sunflower leaves were also used for Cu(II) adsorption in aqueous solutions. The maximum adsorption capacity was determined based on the Langmuir isotherm and was 89.37 mg/g (1.41 mmol/g) [16]. Alomá et al. studied Ni(II) removal from aqueous solutions on sugarcane bagasse by a batch method. Following the experiments, an adsorption capacity of 2 mg/g was determined at 25 °C and pH = 5 on sugarcane bagasse mass [17]. Marin et al. tested maize stalk for Cu(II) and Fe(III) adsorption from synthetic solutions after activation with HCl. The concentration range studied to determine the adsorption capacity of maize stalk was 0.05–0.4 mg/L Cu(II) and Fe(III). Activated maize stalk removed 0.03 mg/g Cu(II) and 0.05 mg/g Fe(III) for 0.4 mg/L synthetic solution with 0.1 g biomaterial [18]. In addition, the ion exchange properties of the biomass resulting from the maize stalk were studied. Initially, study of the adsorption on activated maize stalk was achieved using a solution of 21 metallic elements of 0.02 mg/L. At this concentration, a strong affinity of the activated maize stalk was observed: 91% Mo(VI), 73.7% Pb(II) and 37.3% Sb(III) were adsorbed. The adsorption capacities in a synthetic mixture of Mo(VI), Pb(II) and Sb(III) are presented in Table S1 in the Supplementary Materials [19] in a synthesis of literature data. Davoud et al. used maize stalks for Pb(II) adsorption from aqueous solutions. It has been found that this inexpensive biomaterial can be used for this purpose. The maximum adsorption capacity determined was 5.14 mg/g [20]. Using the batch method, Pb(II) and Cd(II) were retained on sunflower residue. After strain processing, parameters that influence the adsorption process were evaluated. The maximum adsorption capacity was 183 and 68.9 mg/g for Pb(II) and Cd(II), respectively, at optimum batch conditions [21]. Studying the Langmuir equation, the maximum adsorption capacity of wheat straw for Cr(VI) and Cr(III) was determined as being 125.6 and 68.9 mg/g, respectively. Approximate values of adsorption capacity were obtained for corn straw by applying the same experimental model [22]. Štefušová et al. studied the removal of Pb(II) and Cd(II) on wheat straw and rapeseed residues. This study was performed before and after microwave pyrolysis. The best adsorption capacity was determined for rapeseed (31.6 and 83.5 mg/g Cd(II) and Pb(II), respectively) from the studied aqueous solutions [23].

Physical chemical techniques for characterizing the surface of the loaded biomaterial are used to identify the mechanisms of adsorption. FTIR spectroscopy is widely used, most often for the characterization of biomaterials, offering information on the chemical constituents, sample crystallinity and even supramolecular structure [24,25,26]. Recent studies showed the dependence of FTIR peak positions on the maize stalk powder particle size [27], variation in OH group concentrations in the different parts of corn stalk and the adsorption properties and mechanisms [18,27,28,29]. Thus, FTIR studies were used to investigate the removal of iodate with corn stalk and Cr(VI) with bagasse, showing three possible mechanisms, i.e., ion exchange, electrostatic attraction and oxidation reduction reactions in the first case [30,31]. However, to our knowledge, the concept based on using maize stalk for Cu(II) and Fe(III) removal from real mine waters has not been considered yet. The aim and importance of this paper is the proposal to tackle the major problem generated by mining activities, namely the pollution of the environment with metals, by thoroughly evaluating the use of maize-stalk-derived materials for metal adsorption and recovery using batch and pilot studies. The principal novelty consists in developing a pilot study showing the efficient removal of metallic ions with maize-stalk-derived material from genuine water samples polluted as a result of mine exploitation, a scientific first, to the best of our knowledge.

## 2. Materials and Methods

### 2.1. Preparation of Biomaterial

Maize stalk collected after harvesting from a farm was washed after all the leaves were removed and then shredded to 1-mm size in a laboratory using a Matest electric grinder (Macben, Breda, The Netherlands). To improve the adsorption properties, maize stalk was activated in a column using hydrochloric acid 4 M at 1:40 mass per volume ratio.

### 2.2. Chemicals

For the experimental part, standard solutions of 1000 mg/L Pb(NO_3_)_2_, Cu(NO_3_)_2_ and Fe(NO_3_)_3_ in HNO_3_ 0.5 mol/L, 37% HCl, 65% HNO_3_ and KBr solid were purchased from Merck (Darmstadt, Germany).

### 2.3. Characterization Methods and Apparatus

Characterization of maize-stalk-loaded samples was done using a VERTEX 70 FTIR spectrometer (Bruker, Ettlingen, Germany) equipped with a diamond ATR accessory working at 4-cm^−1^ resolution with 128 scans, a scanning electron microscope Quanta FEG 250 (Fei, Eindhoven, The Netherlands) and a STA 409 PC Luxx simultaneous thermogravimeter-differential scanning calorimeter TG/DSC (Netzsch, Selb, Germany) for thermal analysis. FTIR spectra were acquired in quadruplicate, using atmosphere background, the best-quality spectrum, automatically ranked by OPUS software for consideration to be used in the analysis.

### 2.4. Batch Adsorption Methodologies

Initially, selectivity of maize stalk towards metal ions was tested using a 0.04-L solution of 1 mg/L from each metal and 0.5 g of maize stalk activated for 2 h at 175-rpm stirring speed. Moreover, adsorption studies were carried out by combining 0.5 g of shredded maize stalk and 40 mL of metal ion solution at various concentrations at 175-rpm stirring speed. After stirring for 2 h, the supernatant solution was filtered and analyzed using the Atomic Absorption Spectrometry (AAS) technique. Based on this experiment, the adsorption capacity of the maize stalk was determined.

### 2.5. Batch Experimental Procedure for Reutilization and Regeneration of Maize Stalk

Regeneration studies are essential in terms of technological processes as well as economical aspects. It is known that adsorption is controlled by pH, when hydrogen ions from the solution functionalize the cation exchanger centers and replace the metal ions retained by the biomaterial [32]. Reutilization studies were carried out by applying the same methodology described above using 5 mg/L Cu(II) and Fe(III) concentrations in a mixture solution; it should be mentioned that the same sample was reused in five adsorption studies after regeneration. Regeneration studies were done with 200 mL, 3 M HNO_3_ for 0.5 g maize stalk obtained after the adsorption process of metal ions for 1 h at 175-rpm stirring speed.

### 2.6. Laboratory Batch Experimental Procedure for Mine Tailing Pond Decontamination

The performance of maize stalk in the adsorption experiment for mine tailing pond decontamination was evaluated by mixing 40 mL leachate sample, to which 0.5 g of activated maize stalk was added. Thus, the mixture obtained was subjected to mechanical agitation for 2 h at 175 rpm stirring speed, after which the supernatant solution was filtered on quantitative filter paper and then on a membrane filter, namely Whatman 0.45 µm. Supernatant solutions before and after contact with the activated maize stalk were analyzed by AAS.

### 2.7. Analytical Methods

A PinAAcle 900T atomic absorption spectrometer (Perkin Elmer, Norwalk, CT, USA) was used to determine the concentrations of each metal ion in synthetic solution, wastewater samples as well as in leachate samples using flame mode (air-acetylene method). Solutions for detecting the linearity of the analytical method were obtained by diluting certified reference material (CRM) standard solution to achieve a concentration range of 0.1–0.5 mg/L. To this end, a solution of each standard was analyzed and linear regression lines were obtained by representing the emission intensity area in function of concentration. Detection and determination limits of the analytical method for all metals studied are presented in Table 1.

### 2.8. Sampling and Conservation of Mine Wastewater Samples

Mine wastewater was collected in borosilicate glass, after being washed with 10% HNO_3_ solution and rinsed with distilled water. Prior to sampling, borosilicate glass was rinsed with wastewater and the samples taken were acidified for conservation with 65% HNO_3_. In order to avoid precipitation of the metal ions, approximately 1 mL of 65% HNO_3_ was required to obtain a pH in the acidic range. 

### 2.9. Column Adsorption Experiments for Mine Water Treatment

Laboratory pilot installation for mine water treatment consists of a feed tank, peristaltic dosing pump and glass column with internal diameter Ø = 2.5 cm and height (H = 25 cm), which was packed with 3.5 g of activated maize stalk. After swelling in water for 24 h, this was transferred into a column, obtaining material bed of 18 cm height (H_b_). The column experiment was projected in downflow mode with flow rate Q = 0.6 (mL/min). A synthetic polypropylene membrane was installed on the inside as well as on the top of the column to support the biomaterial bed. Thus, with the peristaltic dosing pump, the flow rate of water through the biomaterial bed was established. Based on this, the hydraulic retention time HRT = $\frac{\varphi \cdot H_{b}}{Q}$ (1) was established as being 14 min, where the porosity (*φ)* of the biomaterial was experimentally measured *φ* = 15.6 %. The volume of treated water was also measured with a graduated cylinder. For the laboratory pilot installation used in the mine water treatment, the following operating parameters were determined: the horizontal surface area S $=\frac{\pi }{4}{Ø}^{2}$; S = 4.91 cm^2^ (2); total volume of the column Vt = S × H = 123 cm^3^ (3), working volume Vw = S × H_b_ = 88.2 cm^3^ (4), as well as the velocity of water flow through the biomaterial bed v $=\frac{\pi }{4}{Ø}^{2}$ = 0.07 (m/h) (5). Samples were taken every 12 h, from which Cu(II) and Fe(III) concentrations were determined from inflow and outflow using the AAS technique. Removal efficiency of the metal from the outflow was determined by the following equation: R% $=\frac{C_{i}\;-\;C_{e}}{C_{i}}$ (6). The amount of metal retained at equilibrium (*Q_e_*) in relation to the mass of maize stalk was calculated with $Q_{e}=\frac{\left(C_{i}\;-{\;C}_{e}\right)V}{m}$ (7), where *C_i_* is the initial metal ion concentration used in the adsorption study, *C_e_* represents the concentration in solution after contact with the maize stalk at equilibrium; *V* is the volume of test samples and *m* is the mass of biomaterial used to retain metal ions. 

### 2.10. Column Adsorption Experiments for Synthetic Solution Evaluation

**Figure 1:**
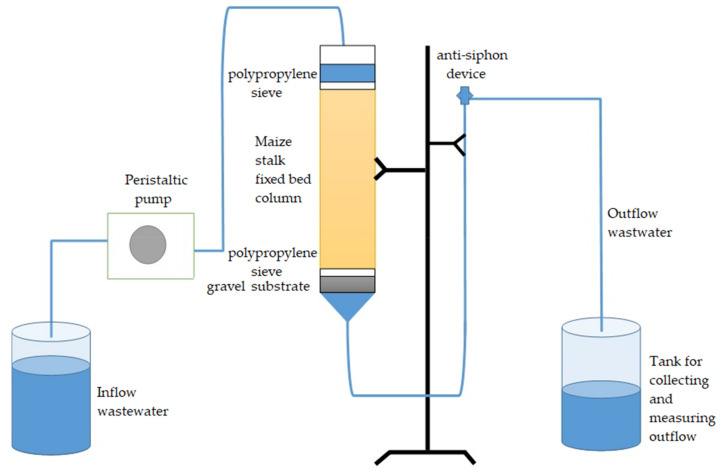
Schematic pilot installation tested in the National Research and Development Institute for Industrial Ecology (ECOIND) laboratory.

The continuous flow adsorption laboratory pilot experiment developed for synthetic solution evaluation was performed in a glass column (Ø = 4.5 cm and H = 50 cm). For this purpose, the previously presented column was packed with a known quantity of material (100 g of activated maize stalk after swelling in water) and hence a biomaterial bed (Hb) of 40 cm was achieved. In order to keep Hb, a polypropylene sieve was installed at the top of the column. Moreover, 4 cm of substrate gravel was introduced inside the bottom of the column. Thus, during the experiment, inflow solution was pumped through the column in downflow mode by a peristaltic pump with a constant volumetric flow Q = 0.6 mL/min, for 11 days, through the biomaterial bed (Figure 1). Our pilot installation was composed of a peristaltic flow controller, a glass column with Vt = 800 cm^3^, Vw = 640 cm^3^, and a tank for collecting and measuring outflow.

## 3. Results

### 3.1. Competitive Studies by Batch Technique

Most articles in the scientific literature are focused on the individual adsorption of metal ions by reutilized systems [33,34,35]. Therefore, the performance of the adsorption system may be mostly evidenced in the multi-element mixture [19,36,37]. For this purpose, the influence of the competitive adsorption in eight metal solutions was studied through the synthetic solution method. Affinity of the maize stalk for metals was determined as follows: 0.059 mg/g Fe(III), 0.055 mg/g Cu(II), 0.040 mg/g Pb(II), 0.032 mg/g Cr(III) while Cd(II), As(V), Mn(II) and Ni(II) have an adsorption capacity of less than 0.001 mg/g. Taking into account this experiment, different matrixes that contain mostly retained metal ions have been studied.

### 3.2. Batch Sorption Laboratory Experiments

**Figure 2:**
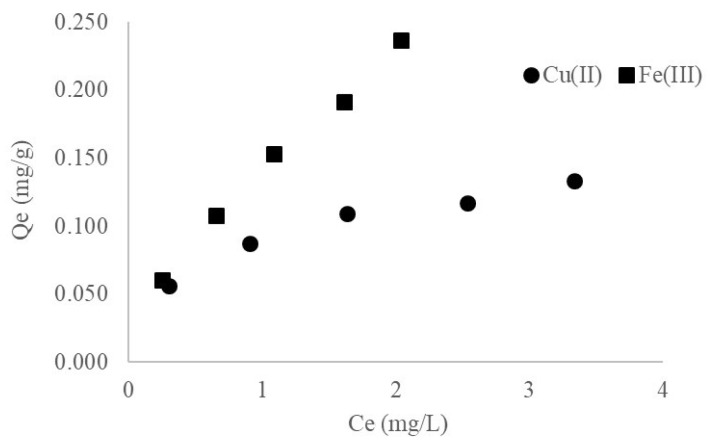
Experimental adsorption isotherm of maize stalk for Cu(II) and Fe(III) removal, i.e., quantity of metallic ions retained at equilibrium (*Qe)* vs. concentration of ions in solution after adsorption on maize stalk at equilibrium *(Ce).* The values represent the average of three replicates, RSD (Relative Standard Deviation) (%) being below 8% for all measurements.

In this assay, the adsorption potential of maize stalk in aqueous synthetic solutions containing Cu(II) and Fe(III) was evaluated. Thus, it was observed that the maize stalk exhibited significant sorption capacities for Fe(III). Maize stalk retained 0.060 to 0.237 mg/g Fe(III), when the initial concentration increased from 1 to 5 mg/L. At the same time, all *Qe* (mg/g) values determined for Cu(II) were lower than those for Fe(III) that is 0.056 to 0.133 mg/g. Overall, this study demonstrates that maize stalk may be used in practical applications for wastewater depollution containing mostly the two metal ions studied, showing very good performance as compared with other wastewater treatment materials, as can be observed in detail in Table S1 in the Supplementary Materials [12,13,14,15,16,17,18,19,20,21,22,23]. All batch adsorption experimental results are presented in Figure 2.

### 3.3. Reutilization of Maize Stalk by Batch Studies

**Figure 3:**
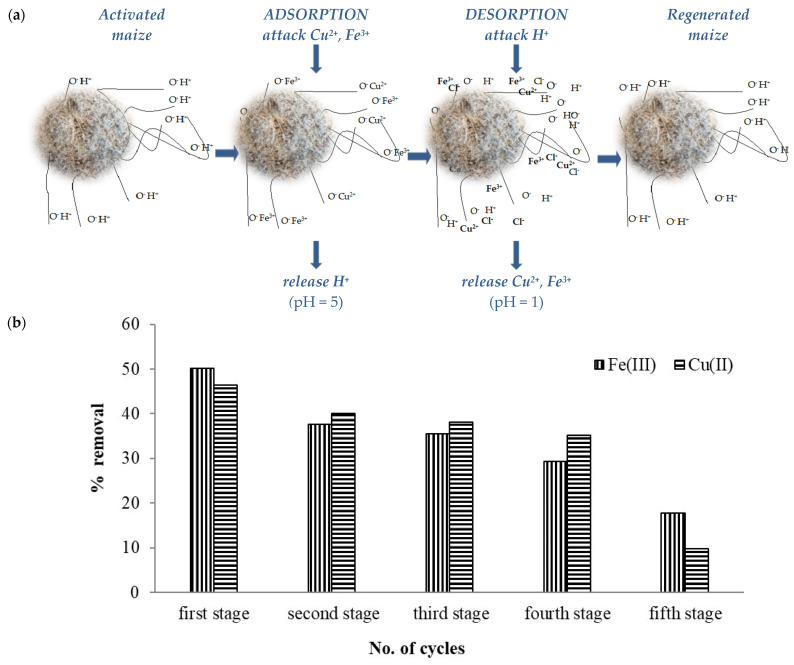
(**a**) Schematic representation of adsorption/desorption mechanism of Cu(II) and Fe(III) on maize stalk material. (**b**) Reutilization behavior of maize stalk in adsorption process. The values represent the average of three replicates, RSD (%) being below 8% for all measurements.

At present, the reutilization of vegetable materials in the adsorption process represents a significant benefit not only from an economic point of view but also for environmental protection [17,38,39]. Taking into consideration these aspects, the following experiment was performed to evaluate the reutilization of maize stalk. After the first utilization of the maize stalk mass, a desorption stage was conducted. The desorption process was applied after each adsorption stage. The adsorption/desorption mechanism of Cu(II) and Fe(III) on shredded maize stalk (1 mm) for the new reutilization stage is presented in Figure 3a.

From the results achieved, it was observed that, from one stage to another, the adsorption capacity of maize stalk decreased slightly. For the Fe(III) presence, the removal percentages on maize stalk were as follows: up to 50.09% (first stage) > 37.7% (second stage) > 35.6% (third stage) > 29.3% (forth stage) and 17.8% in the last stage. At the same time, Cu(II) followed an identical adsorption trend on maize stalk mass, from 46% (first stage) > 40.3% (second stage) > 38.1% (third stage) > 35.2% (forth stage) to 9.7% reached at the final stage, a low removal percentile compared with the previous stage. Reutilization behaviors are illustrated in Figure 3b.

### 3.4. Efficient Cu(II) and Fe(III) Removal from Leachate Tailing Samples by Batch Technique 

The tailing samples studied in this experiment consist of a sterile paste deposited in the settling pond, acquired by processing polymetallic gold and silver ore. Tailing pond samples were collected diagonally from the same area at depths in the range of 20–40 cm and subsequently were transferred in plastic bags to the laboratory, where they were dried, sieved and analyzed. For this purpose, each sample was leached as follows: 20 g of dried tailing was quantitatively transferred to a 200-mL Erlenmeyer flask made from PYREX^®^ glass (brought to the mark with ultra-pure water) and stirred for 24 h at 100-rpm scanning speed in a horizontal shaker. In this respect, after applying the leachate test, we obtained the following initial concentrations: 25 mg/L Cu(II) and 100 mg/L for Fe(III) in sample 1 (S1). For sample 2 (S2), 75 mg/L Cu(II) and 25 mg/L Fe(III) were obtained; for sample 3 (S3), the concentrations detected were 80 mg/L Cu(II) and 290 mg/L Fe(III). Taking into consideration sample 4 (S4), the leachate concentrations determined were 45 mg/L Cu(II) and 210 mg/L Fe(III). Rapid availability was observed for sample 5 (S5), for which values of 126 mg/L Cu(II) and 705 mg/L Fe(III) were obtained. At the same time, for sample 6 (S6), the results were 54 mg/L Cu(II) and 501 mg/L Fe(III). 

**Figure 4:**
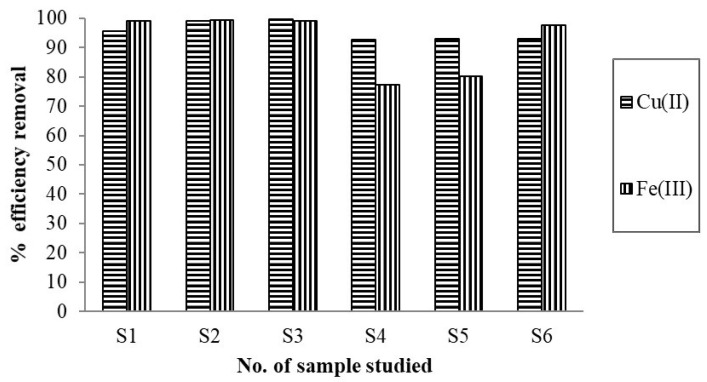
Percentages of efficiently removed Cu(II) and Fe(III) onto maize stalk mass.

High concentrations of available metals obtained by applying the leachate test may exert a negative effect on the environment over time, and thus, in this study, the issue of unconventional technology and additionally of specific recovery to increase the economic efficiency of metal production was analyzed. To achieve this goal, depolluted samples were obtained using a batch laboratory procedure applying the methodology described in Section 2.6. Initially, for S1, the maximum adsorption capacity of 7.66 mg/g was obtained for Fe(III) and 1.98 mg/g for Cu(II). The experimental results for S2 revealed maximum capacities of the maize stalk as follows: 1.98 mg/g Fe(III) and 5.96 mg/g Cu(II) removed. For S3, the adsorption capacity determined for Fe(III) was 23.2 mg/g, and for Cu(II), it was 6.35 mg/g. Maize stalk mass removed from S4 was 15.6 mg/g Fe(III) and 2.78 mg/g Cu(II). In addition, efficient removal in the biomaterial mass was demonstrated for S5 (52.4 mg/g Fe(III) and 8.10 mg/g Cu(II)) as well as for S6 (37.3 mg/g Fe(III) and 4.22 mg/g Cu(II)), respectively. Moreover, efficient removal during the batch adsorption experiment is demonstrated in Figure 4. From another point of view, the percentages removed in the maize stalk mass were very high, ranging between 92.6% and 99.1% for Cu(II) and 77.3% and 99.3% for Fe(III), which suggests the quantitative retention of cationic species in all samples evaluated.

To conclude, a high metal depollution capacity may be obtained using this new concept that presents the benefit of reduced in situ costs compared to the conventional methods for metal pollutant removal, especially for Cu(II) and Fe(III) in the field of mining activities. The values represent the average of three replicates and RSD (%) being below 8% for all measurements. 

### 3.5. Applications of Maize Stalk in Column Experiment

#### 3.5.1. Efficient Cu(II) and Fe(III) Removal from Mine Wastewater by Column Studies

**Figure 5:**
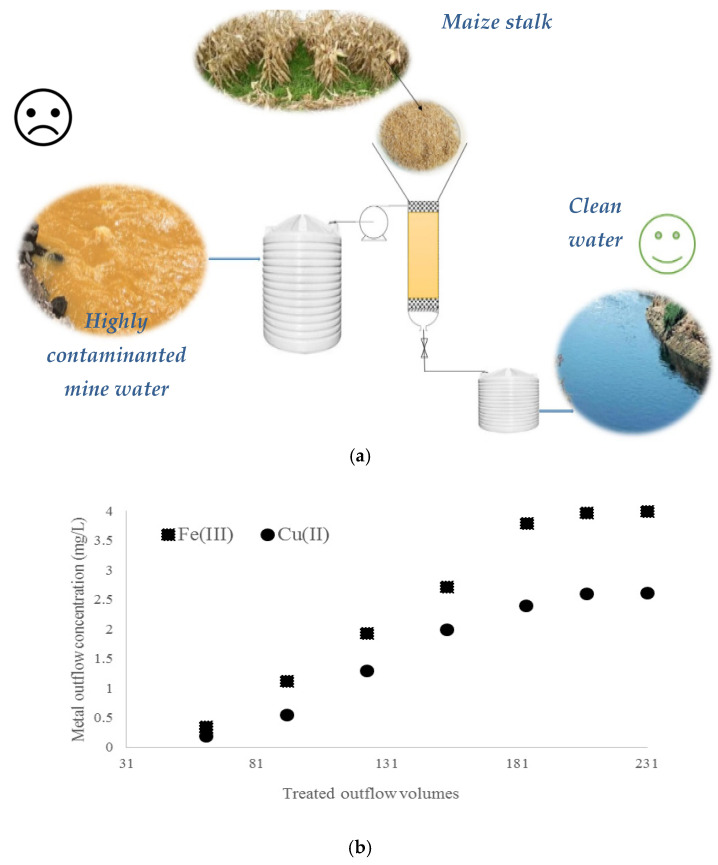

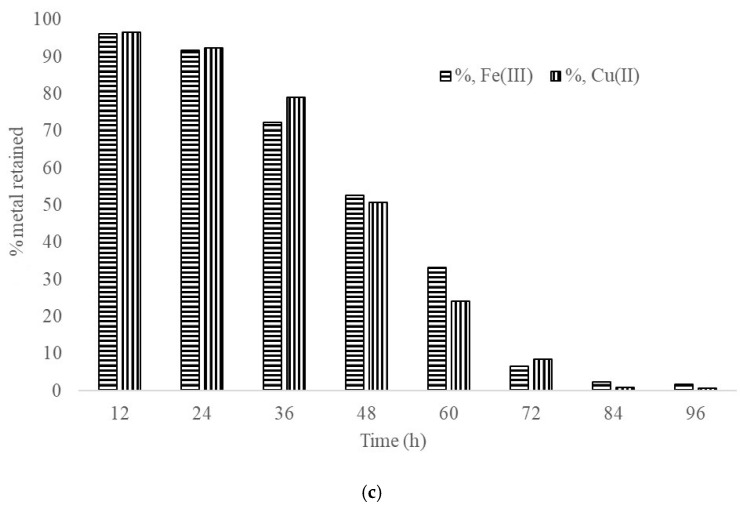
(**a**) Schematic of mine water treatment process. (**b**) Adsorption of Cu(II) and Fe(III) in relation to treated outflow volumes. (**c**) Efficient removal of Cu(II) and Fe(III) in continuous flow mode. The values represent the average of three replicates and RSD (%) being below 8% for all measurements.

Until now, the removal of metals using modified biomass resulting from the harvesting of agricultural crops is mostly applied only at the batch experimental stage. Experimental engineering applications which have been very little tested up until now are subjected to analysis in this study. It is known that water from mining is a complex matrix, rendering it an interesting biomaterial to use in developing technologies with the main goal of the removal of pollutants from the environment. The applicability of maize stalk in Cu(II) and Fe(III) adsorption has been tested through the treatment of mine water obtained after conventional treatment with Ca(OH)_2_. For this purpose, swollen maize stalk was tested under the conditions described in Section 2.9, where 3000 mL mine water was treated in a column experiment. A fixed-bed schematic representation of mine water treatment based on maize stalk is presented in Figure 5a. As one may observe from Figure 5b, metal outflow adsorption decreased in the biomaterial bed with increasing treated outflow volume. In this case, a possible explanation could be the decrease in the number of active sites available for initial adsorption, and it is difficult to engage fewer accessible sites. Moreover, we observed a saturation plateau in the biomaterial bed when available groups or macroporous structures are loaded [40,41]. 

Moreover, the rapid adsorption of metals was observed when monitoring in continuous flow mode for 60 h (Figure 5c). Throughout the experiment, maize stalk demonstrated the capacity to remove up to 24.2% Cu(II) and 33.3% Fe(III) from inflow polluted mine water with 2.625 mg/L Cu(II) and 4.070 mg/L Fe(III) concentrations.

#### 3.5.2. Column Experiment Using Solutions of Cu(II) and Pb(II)

**Figure 6:**
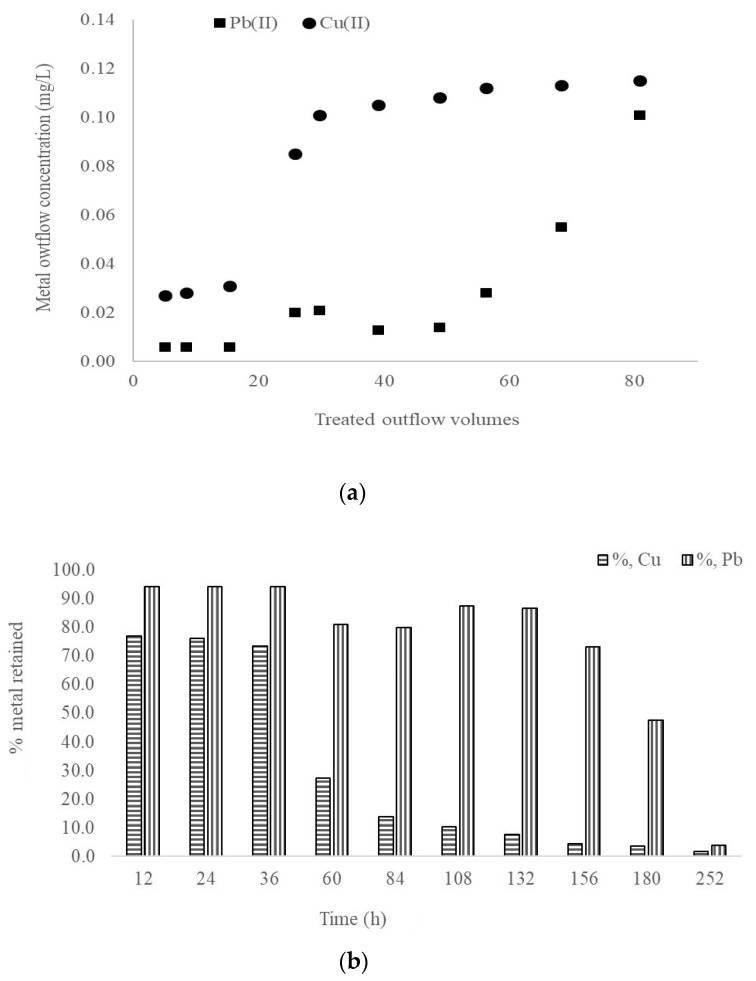
(**a**) Adsorption of Cu(II) and Pb(II) reported for treated outflow volumes. (**b**) Efficient removal of Cu(II) and Pb(II) in continuous flow mode. The values represent the average of three replicates and RSD (%) being below 8% for all measurements.

Starting from the first pilot experiment, where the biomaterial bed was saturated in approximately 72 h, in this experiment, based on the pilot installation developed in Figure 1, the charge of the biomaterial bed inside the column was increased and the metal concentration from inflow was decreased in order to evaluate maize capacity over a long time period in continuous flow mode. Moreover, the volume of water treatment was experimentally determined by measuring the water volume retained in the porosity of the biomaterial bed. In addition, the Cu(II) and Pb(II) influence from the synthetic solution on the biomaterial bed was studied at pH 2.5 ± 0.2. Samples were collected after 12 h in the first 2 days, and then after 24 h. The removal means of Cu(II) and Pb(II) were higher on the initial days of testing. Following the experimental monitoring after 36 h of continuous flow treatment, the percentages obtained from inflow were 73.5% for Cu(II) and 94.28% Pb(II) on the biomaterial bed, achieving saturation after 252 h of study (Figure 6a).

Another important finding is that, if we refer to the total volumes of water treated, taking into account the porosity fraction of the biomaterial bed [42], up to 68 volumes of water with 0.105 mg/L Pb(II) content may be treated and up to 15 volumes of inflow water with 0.117 mg/L Cu(II), showing the increased efficiency of the pilot experiment (Figure 6b).

Taking into account the end of the column experiment, the saturated material was regenerated with 300 mL HNO_3_ 3 M, which was passed in up flow mode through the column using the same flow volume. The regenerated agent was sufficient to bio-recycle approximately 93% Cu(II) and 89% Pb(II) retained in the biomaterial bed from the column. 

In conclusion, the relevance of using this innovative concept ranges from environmental protection to long-term economic advantages due to the specific recovery of metals retained and the possibility of biomaterial reutilization.

### 3.6. Maize Stalk Characterization after Mine Water Treatment

#### 3.6.1. FTIR-ATR Studies

**Figure 7:**
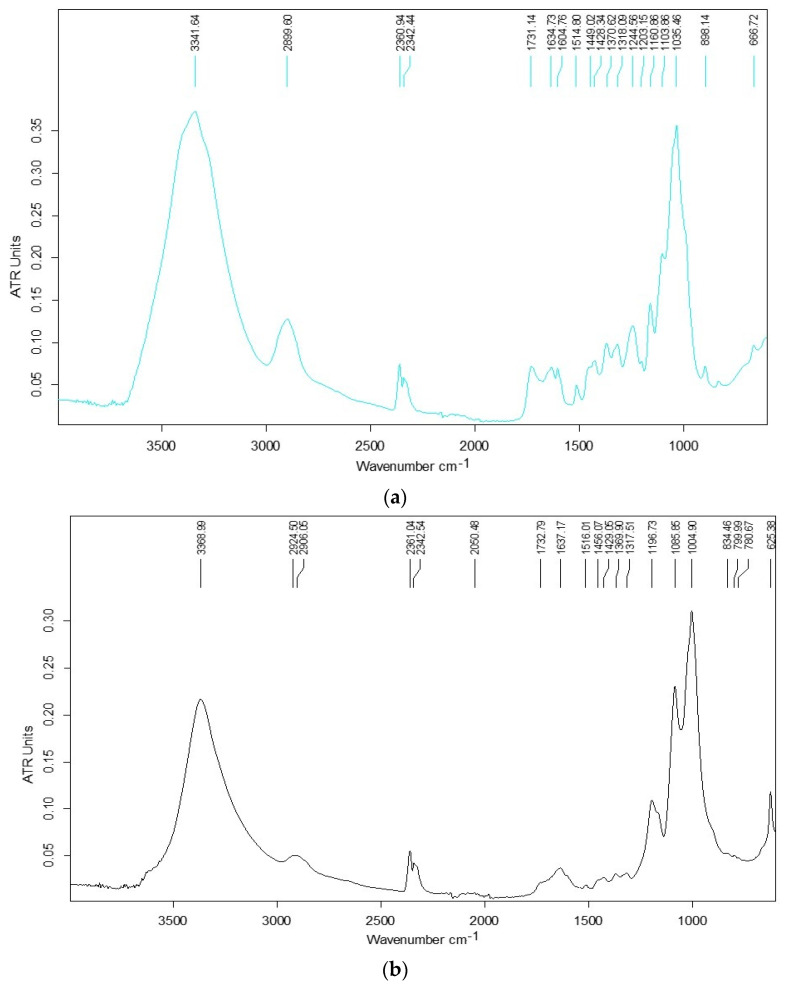
FTIR-ATR spectra of maize stalk used in sorption of Cu(II) and Fe(III) (**a**) from mine water and (**b**) from leachate solutions.

Functional groups of bio-sorbents, including alcohol, aldehyde, ketone, carboxylic acid, phenols and ether linkages, bind metal ions by using an electron pair to form complexes in the solution [43]. Carboxylic and phenolic groups are the main active sites involved in metal removal [44]. FTIR-ATR analysis of maize stalk showed characteristic signals at 3340.71 cm^−1^ specific of the –OH functional group, and other characteristic peaks were observed at 2899.08, 1712.57, 1514.32, 1369.51 and 1034.36 cm^−1^, emphasizing the presence of ν CH, ν C=O, ν C=C, δ C–H and ν C–O groups, as determined in our previous research [18]. In Figure 7, the FTIR-ATR spectra of maize stalk loaded with leachate solution and mine water are shown, and the main characteristic frequencies are shown in Table 2. Significant spectral differences are observed for maize stalk treated with tailing solution, e.g., large band displacements, in the order of tens of cm^-1^ in the region of 1000–1250 cm^−1^, characteristic of C–O–C vibrations in cellulose/hemicellulose. In this case, an important decrease in the bands’ intensity compared to the control sample is observed; essentially, in the tailing solution, ion adsorption onto the surface attenuates the signal of the biomaterial, so a general decrease in peak intensity was observed.

The position, intensity and shape of the maize stalk hydroxyl band changes after treatment with leachate and mine solutions, resulting in a different hydrogen bond interaction pattern. Moreover, the C=O group shifts from 1712 to 1731 and 1732 cm^−1^, respectively, showing the interaction with the metallic ions. The use of FTIR investigation techniques proved useful for providing complete information on the interaction mechanism of maize stalk with Cu(II) and Fe(III) by emphasizing the contribution of polar groups to the retention of metal ions and the minimal contribution of non-polar fragments to the adsorption process.

#### 3.6.2. Thermal Analysis 

**Figure 8:**
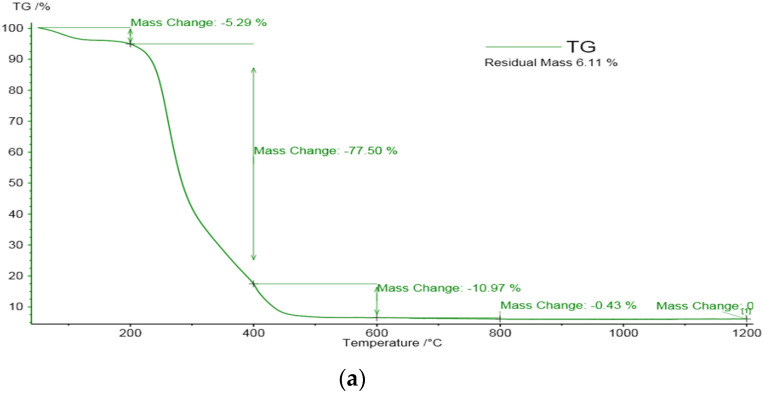

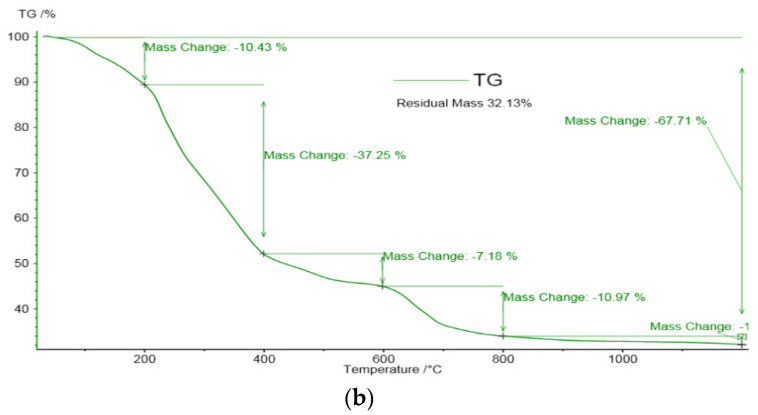
Thermal decomposition of maize stalk after loading with metal ions from (**a**) mine water and (**b**) from leachate solutions.

The adsorption of Cu(II) and Fe(III) ions in maize stalk after mine water treatment and from leachate tailing solutions was evaluated by thermal analysis. The thermal decomposition (TG) behavior of activated maize stalk was assessed in previous research [18]. Using TG, a variation in weight loss depending on the temperature range was observed. In the first pyrolysis stage, TG curves showed up to 5.29% and 10.5% weight loss in the 30–200 °C temperature range (Figure 8a,b) from the solid phases studied, which is associated with water molecules being retained by metal ions trough ionic bonds. In the next stage, significant weight loss in the 200–400 °C temperature range may be attributed firstly to hemicellulose and secondly to cellulose pyrolysis. The lower weight mass loss of 32.3% for maize stalk loaded with tailing solution suggests the occurrence of metal ion retention in the biomaterial mass, which influenced the stabilization of the hemicellulose/cellulose structure.

#### 3.6.3. Scanning Electron Microscopy (SEM)

**Figure 9:**
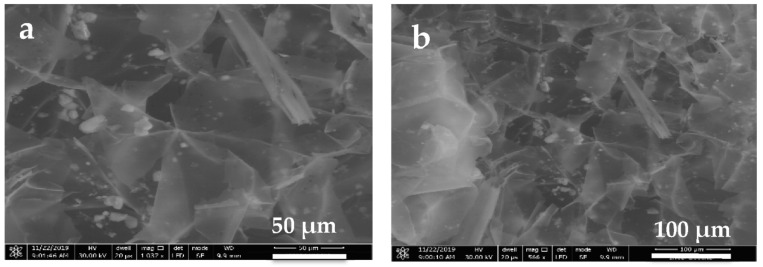

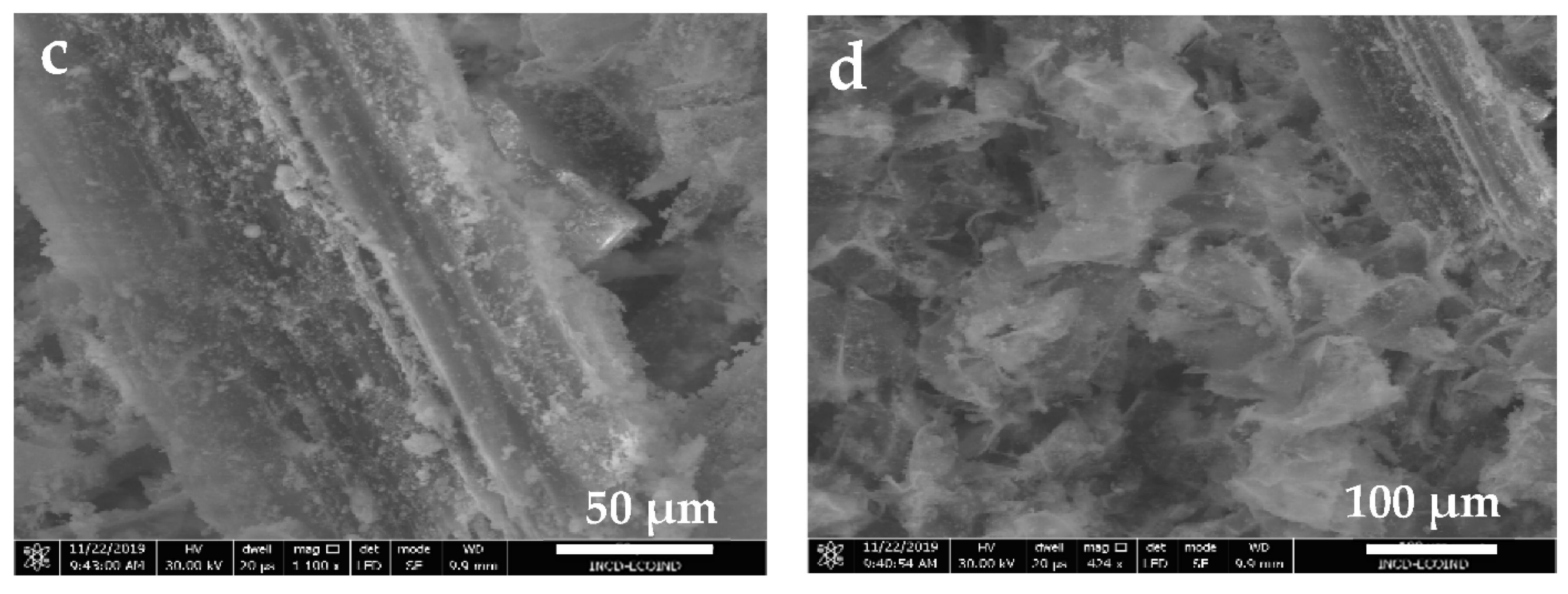
SEM micrographs of metal-loaded maize stalk mass at 50 and 100 µm resolution, respectively after: (**a**,**b**) mine water adsorption; (**c**,**d**) leachate solution adsorption.

The metal adsorption from the environmental matrix in maize stalk mass was evaluated using SEM-specific images. In the literature, morphological evaluation found that acrylonitrile-modified corn stalk mass became homogeneous and well-ordered with the increase of the porosity of the material (adsorption capacity was 3.76 times higher than that of the unmodified material) [12]. Song et al. characterized raw rice straw and mercapto-grafted rice straw using SEM images. For raw rice straw, an uneven surface with prominent globular formations was observed. Moreover, the surface morphology of mercapto-grafted rice straw was different compared to the base material, in which a fractured and much rougher surface was observed. The SEM image showed changes in the biomaterial, where the rough and irregular surface led to an increase in the adsorption capacity of Hg(II) [39]. In our case, as already was observed in a previous study, a more porous surface was highlighted that increases the adsorption properties of this biomaterial after acid treatment [18]. Moreover, acid treatment of maize stalk contributes to increased porosity and also to elimination of the deposits, the activation of the functional groups existing in the material’s structure. Therefore, after the adsorption experiments, the complete coverage of porous structure was observed (Figure 9) and this behavior is more evident for the maize stalk mass used in the green depollution tailing experiments (Figure 9a,d). 

## 4. Conclusions

The preparation of maize-stalk-derived materials for water purification applications is described. The paper focuses on specific removal of Cu(II) and Fe(III) from real, highly polluted tailing pond and mine wastewater samples. Additionally, Cu(II) and Pb(II) from synthetic solutions were monitored to test the derived materials for metal adsorption and recovery. FTIR-ATR, TG and SEM techniques were used to characterize the interaction between maize stalk and test solution samples. With this aim, the adsorption capacity of maize stalk material towards Fe^3+^ and Cu^2+^ was measured, revealing high performance for the removal Fe^3+^. In addition, the prepared materials demonstrated very good efficiency for the depollution of wastewater affected by mine exploitation. The adsorption capacity of maize stalk material was tested on a mechanically stable granulation suitable for use in a wide range of applications. Shredded maize stalk with a 1 mm particle size was activated in a fixed-bed column with HCl 4 M and subsequent batch and column laboratory experiments were carried out. The batch adsorption capacity for Fe(III) was 1.8 times higher than for Cu(II) in batch conditions. Moreover, in multielement solutions, Fe(III) is better retained by maize stalk than Cu(II). In addition, very good results were obtained when maize stalk was tested for depollution of mining sites consisting of waste tailings together with wastewater resulting from mine exploitation. The investigation with the ATR-FTIR techniques provided useful information on the process for evaluating the maize stalk exchange mechanism with metallic ions. Data obtained from pilot experiments may be used for the purposes of water depollution. Moreover, in order to implement it, the maize-material-based water depollution technology requires a simple preparation procedure and installation. Furthermore, our research results are in line with the principles of green technology and the estimated cost of environmental depollution is very low compared with conventional technologies.

## Figures and Tables

**Table 1:** Linearity parameters of the analytical method.

Metal Ions	λ (nm)	Linear Regression Equation	Correlation Coefficient	Detection Limit (mg/L)	Determination Limit (mg/L)
Cu(II)	324.75	y = 0.1252x + 0.0007	0.9993	0.0010	0.0035
Fe(III)	248.33	y = 0.0854x − 0.0004	0.9997	0.0020	0.0065
Pb(II)	283.31	y = 0.0220x − 0.0021	0.9996	0.0012	0.0040

**Table 2:** Vibration frequencies of maize stalk post loading with Cu(II) and Fe(III).

Functional Groups	Maize Stalk Loaded with Metals from Mine Water (cm^−1^)	Maize Stalk Loaded with Metals from Tailing Solution (cm^−1^)
ν OH	3341.64	3368.99
ν CH	2899.60	2906.05
ν C=O	1731.14	1732.79
ν C=C	1514.80	1516.01
δ C–H	1370.62	1369.90
ν C–O	1036.46	1004.00

## Data Availability

Not applicable.

## Supplementary Materials

The following are available online at https://www.mdpi.com/1996-1944/14/4/956/s1, Table S1: Study of adsorption properties of cellulosic material for metal removal from aqueous solutions.
