# Supplementary material for: Maize Stalk Material for On-Site Treatment of Highly Polluted Leachate and Mine Wastewater
Source: Materials (Basel). 2021 Feb 18;14(4):956. doi: 10.3390/ma14040956 (PMC7922871; doi:10.3390/ma14040956)
Supplement: Supplementary file 1 [file materials-14-00956-s001.pdf]

materials

MDPI

Supplementary Material

Maize Stalk Material for On-Site Treatment of Highly Polluted Leachate and Mine Wastewater

Nicoleta Mirela Marin *, Laurentiu Dinu, Ioana Stanculescu *, Nicolae Ionut Cristea and Alexandra Ioana Ionescu

Table S1. Study of adsorption properties of cellulosic material for metal removal from aqueous solutions.

Raw or Modified Cellulosic Residue			Metals	Adsorption Capacity (mg/g)	Reference
Corn	stalk	acrylonitrile treatment	Cd(II)	12.73	[1]
		graft copolymerization	Cd(II)	21.37	[2]
		etherification	Cd(II)	12.73	[3]
			Pb(II)	31.80	
			Cu(II)	9.34	
		grafting	Cd(II)	22.17	
		activation with HCl	Cu(II)	0.03	[4]
			Fe(III)	0.05	
			Sb(III)	0.45	[5]
			Mo(VI)	0.40	
			Pb(II)	0.30	
		raw	Pb(II)	5.14	[6]
	straw		Cr(VI)	87.4	[7]
			Cr(III)	62.3	
	cob activated with organic acid		Cd(II)	8.89	[8]
Sunflower		leaves	Cu(II)	89.37	[9]
		stalk	Cd(II)	68.90	[10]
			Pb(II)	183	
Sugarcane bagasse			Ni(II)	2.00	[11]
Wheat straw		raw	Cr(VI)	126	[7]
			Cr(III)	69	
			Cd(II)	5.50	[12]
			Pb(II)	14.0	
		microwave pyrolyzed	Cd(II)	10.5	
			Pb(II)	33.5	
Rapeseed		raw	Cd(II)	31.6	
			Pb(II)	83.5	
		microwave pyrolyzed	Cd(II)	4.80	
			Pb(II)	26.2	

References

1. Zheng, L.; Dang, Z.; Yi, X.; Zhang, H. Equilibrium and kinetic studies of adsorption of Cd(II) from aqueous solution using modified corn stalk. *J. Hazard. Mater.* **2010**, *176*, 650–656, doi:10.1016/j.jhazmat.2009.11.081.
2. Zheng, L.; Zhu, C.; Dang, Z.; Zhang, H.; Yi, X.; Liu, C. Preparation of cellulose derived from corn stalk and its application for cadmium ion adsorption from aqueous solution. *Carbohydr. Polym.* **2012**, *90*, 1008–1015, doi:10.1016/j.carbpol.2012.06.035.
3. Zheng, L.; Lu, G.; CAO, W.; Peng, D.; Zhu, C.; Lin, F.; Long, T.; Yalan, Z.; Zhoulin, L.; Yi, X. Modified Cellulose of Agricultural Residue Used for Removal of Heavy Metals and Oil: Technologies and Applications. *Polymer science: Research advances, practical applications and educational aspects* **2016**.

4. Marin, N.M.; Batrinescu, G.; Stanculescu, I.; Constantin, L.A.; Cristea, I.; Ionescu, I.; Catrina, G.A. Experimental Model for Cu(II) and Fe(III) Adsorption from Synthetic Solutions Based on Maize Stalk. *Rev. Chim*. **2020**, *71*, 355-367.
5. Marin, N.M.; Pascu, L.F.; Stanculescu, I.; Iordache, O.; Jianu, D.; Petrescu, L.; Badea, I.A. Maize Stalk as Natural Ion Exchanger for Hazardous Pollutants. *Rev. de Chim*. **2017**, *68*, 1726–1731, doi:10.37358/rc.17.8.5753.
6. Davoud, B.; Fredos, K.M.; Hossein, A. Adsorption Isotherm Studies of Pb(ll) Ions from Aqueous Solutions by Maize Stalks as a Cheap Biosorbent. *J. Sci. Eng. Res*. **2016**, *3*, 85–91.
7. Chen, Y.; Chen, Q.; Zhao, H.; Dang, J.; Jin, R.; Zhao, W.; Li, Y. Wheat Straws and Corn Straws as Adsorbents for the Removal of Cr (VI) and Cr (III) from Aqueous Solution: Kinetics, Isotherm, and Mechanism. *ACS Omega* **2020**, *5*, 6003–6009.
8. Vaughan, T.; Seo, C.W.; Marshall, W.E. Removal of selected metal ions from aqueous solution using modified corn cobs. *Bioresour. Technol*. **2001**, *78*, 133–139.
9. Benaïssa, H.; Elouchdi, M. Removal of copper ions from aqueous solutions by dried sunflower leaves. Chem. Eng. Process. *Process. Intensif*. **2007**, *46*, 614–622, doi:10.1016/j.cep.2006.08.006.
10. Jalali, M.; Aboulghazi, F. Sunflower stalk, an agricultural waste, as an adsorbent for the removal of lead and cadmium from aqueous solutions. *J. Mater. Cycles Waste Manag*. **2013**, *15*, 548–555, doi:10.1007/s10163-012-0096-3.
11. Alomá; I.; Martín-Lara, M.; Rodríguez, I.; Blázquez, G.; Calero, M. Removal of nickel(II) ions from aqueous solutions by bioadsorption on sugarcane bagasse. *Taiwan Inst. Chem. Eng*. **2012**, *43*, 275–281.
12. Štefušová, K.; Lovás, M.; Zubrik, A.; Matik, M.; Václavíková, M. Removal of Cd2+ and Pb2+ from aqueous solutions using bio-char residues. *Nova Biotechnol. Chim*. **2012**, *11*, 139–146.
